# The Central Role of Inflammation Associated with Checkpoint Inhibitor Treatments

**DOI:** 10.1155/2018/4625472

**Published:** 2018-10-17

**Authors:** Cristina Vajaitu, Carmen Cristina Draghici, Iulia Solomon, Cristina Victoria Lisievici, Alexandra Victoria Popa, Mihai Lupu, Constantin Caruntu, Maria Magdalena Constantin, Vlad Mihai Voiculescu

**Affiliations:** ^1^Department of Dermatology, Elias Emergency University Hospital, Bucharest, Romania; ^2^Department of Dermatology, Medas Medical Center Bucharest, Romania; ^3^Carol Davila University of Medicine and Pharmacy, Bucharest, Romania; ^4^Department of Dermatology, Prof. N. Paulescu National Institute of Diabetes, Nutrition and Metabolic Diseases, Bucharest, Romania; ^5^2nd Department of Dermatology, Colentina Clinical Hospital, Bucharest, Romania

## Abstract

An important function of the immune system is its ability to differentiate between healthy cells in the organism and “foreign” cells, allowing the latest to be attacked and the first ones to be conserved. The most important molecules in this process are considered to be checkpoint inhibitors. This review is focused on the association between cancer and inflammation, underlying the mechanisms of action of monoclonal antibodies that are targeting checkpoint inhibitors: ipilimumab against cytotoxic T-lymphocyte-associated protein 4 (CTLA-4) and pembrolizumab and nivolumab against programmed cell death protein 1 (PD-1), their indications for treatment, and side effects. Presence of antibodies against checkpoint inhibitors shows promising results in the clinical trials in patients with types of cancer difficult to treat until now such as melanoma, non-small-cell lung cancer (NSCLC), and renal cell carcinoma, offering an increase in the overall survival rate, response rate, and progression-free rate. Resistance is now observed to emerge in patients treated with this therapy, showing the need for more studies in order to design a biomarker that will predict the type of response to immunotherapy.

## 1. Introduction

The immune system plays an important role in controlling malignant flare-ups, in this way abolishing cancer. In patients who developed cancer, there are multiple immune suppression mechanisms that prevent the development of a competent antitumor response. Current advances revealed that antibodies against negative immunologic regulators such as checkpoint inhibitors can have success in treating a wide variety of malignancies, both solid and hematologic. Even though cancer has a major impact around the globe, an increase in the survival rate of the people suffering from cancer was registered, due to the development of these new therapeutic approaches [1]. Checkpoint inhibitors have shown clinical efficacy in several solid malignancies including melanoma, renal cell carcinoma, NSCLC, bladder cancer, head and neck squamous cell carcinoma, Merkel cell carcinoma, and hematologic malignancies such as Hodgkin lymphoma. A particularly successful effect was observed in melanoma, nowadays being approved both checkpoint inhibitors anti-PD-1 or PD-L1 (nivolumab, pembrolizumab) and anti-CTLA-4 (ipilimumab), as monotherapy or in association between them [1–3]. Even though melanoma accounts only for 4% of all skin cancers, it is responsible for 80% of the deaths due to malignancies of the skin [4]. It is one of the cancer types that benefit the most from immunotherapy, especially in advanced stages. Melanoma appearance is considered to be closely bound to UV exposure [5] and stress. In vitro, the association with stress was explained by the observation that high level of cortisone, epinephrine, and norepinephrine in cellular environment leads to an increase of murine B16F10 melanoma cells [6].

## 2. Inflammatory Response in Cancer

Inflammation in cancer is sustained by a series of immune and nonimmune cells and the molecules that are secreted by these.

Malignant cells secrete a wide variety of molecules such as cytokines and chemokines, which attract a diverse population of leukocytes (neutrophils, dendritic cells, macrophages, mast cells, and lymphocytes). These will in turn produce an array of molecules that contribute to the fight against malign cells, these molecules being TNF-alpha, interleukins, interferons, and several other agents that are able to damage the plasmatic membranes of the cancerous cells.

There are several immune cells that are in the tumorigenesis process and are endowed with protumor functions. Macrophages associated with the tumor have opposite roles in the malign environment: they can kill the cancerous cells after being activated by IL-2, interferon, and IL-12, but also they can secrete angiogenic and lymphangiogenic growth factors that enable the tumor cells to proliferate [7].

The mechanism of attracting immune cells to the cancerous environment is explained trough the fact that around 15% of all cancers arise from areas that were or still are subjected to infection [8]. Persistent infections will lead to a chronic inflammation. The immune cells will produce reactive oxygen and nitrogen species. These will induce permanent genetic alterations of the proliferating cells by producing a mutagenic agent called peroxynitrite.

It can be concluded that inflammatory cells play an important role in cancer development. In the first stages of cancer, these cells promote tumor growth by producing growth and survival factors that will enable angiogenesis and lymphangiogenesis. In the later stages, inflammatory cells can produce chemokines that are able to help the malignant spread, thus leading to metastasis. On the other site, the inflammatory cells will trigger the immune response which will be counterproductive for the tumor development [9].

Inflammation and tumor generation have a close causal relationship between them. On one side, an inflammatory state can precede a cancerous growth, but on the other side, a malignant transformation can lead to inflammation in the cellular environment which will maintain the cancerous state. Accumulation and production of inflammatory molecules such as cytokines over a long period of time will lead to an immunosuppressant state which is linked to tumor progression [10].

## 3. Types of Immunotherapies

The era of immunotherapy in cancer dates back to the late 19^th^ century. It all started with the idea of William Coley, an American surgeon, who inoculated bacteria in a sarcoma in order to shrink the tumor. His idea was based on the fact that bacteria will be able to trigger an immune response, which will eventually lead to a sustained antitumor immune response.

For most of the 20^th^ century, scientists struggled to convert Coley's observations into an effective cancer treatment. This struggle lead to success; thus, nowadays there is a variety of immunotherapies that are making their way to the clinic. Immunotherapies can be divided into four categories: nonspecific immune stimulation, adoptive cell transfer, checkpoint inhibitors, and vaccination.

### 3.1. Nonspecific Immune Stimulation

In principle, in vivo nonspecific immune stimulation leads to a general increase of the immune response. Inoculated molecules activate the antigen-presenting cells (APCs) by binding to specific membrane receptors. Activated APCs stimulate other immune cells such as T cells, the principal antitumor cells. In order to be fully activated, T cells need the presence of some cytokines such as interferon alpha (IFN-alpha) and interleukin 2 (IL-2). These cytokines have already gained their role in cancer treatments [11].

Nonspecific immune stimulation can be achieved by BCG (bacillus Calmette-Guerin) vaccination. This can give a boost of the immune system that can lead to an increase in cancerous fight. The mechanism is based on the fact that attenuated bacteria can lead to inflammation, attracting more immune cells in the tumor microenvironment. The increased number will raise the chances of attacking the cancerous cells [12].

### 3.2. Adoptive Cell Transfer

Adoptive cell transfer is a type of in vitro manipulated therapy, which uses cytotoxic T cells isolated from the patient. These extracted cells are activated in vitro in order to be able to specifically target the cancerous cells.

T cells can be taken either directly from the tumor or from the blood. The advantage of the first procedure is the fact that immune cells are already exposed to specific antigens from the tumor microenvironment. Then, the harvested cells are activated using cytokines and multiplied before being transferred into the patient [13].

### 3.3. Checkpoint Inhibitors

Nonspecific immunity can also be achieved by removing immune checkpoint inhibitors. These inhibitors normally dampen down the immune response to prevent collateral damage to healthy tissue. In order to restore the active antitumoral immune response, scientists need to remove some of these inhibitors to make the immune response stronger. This is the case of ipilimumab, an antibody which can target a blockade molecule called CTLA-4 [1].

### 3.4. Vaccination

In comparison to the BCG vaccine that induces a general immune response, viral vaccines can be used to direct immune cells to target the malign environment. An example of this type of vaccine is an attenuated version of herpes simplex virus adapted to induce an immune-stimulating factor at the tumor site. This therapy is suitable for melanoma or head and neck carcinoma. T-VEC (talimogene laherparepvec) is a genetically engineered form of herpes simplex virus type-1, having removed two genes in order to be unable to replicate intracellular in normal cells, while maintaining the cytolytic activity against cancerous cells [14, 15]. The multiplication inside cancerous cells leads to their burst (oncolytic effect) and triggers a systemic anticancerous immune response.

In nontumoral, but viral infected cells, viral replication takes place when ICP34.5, a gene that gives the herpes virus the propriety of neurovirulence, forms a complex with low levels of proliferating cell nuclear antigen (PCNA), a protein which is involved in DNA (deoxyribonucleic acid) replication and repair. In cancerous cells, the level of PCNA is high, so the herpes virus is able to replicate without the need of ICP34.5 gene [16]. The most recent clinical trials used HSV vaccine with an additional modification—the insertion of a gene which produces GM-CSF (granulocyte-macrophage colony-stimulating factor), which will improve the anticancerous effects by increasing the stimulation and recruitment of dendritic cells [17–19].

Another type of vaccination is with irradiated autologous cancerous cells engineered to secrete growth factors such as GM-CSF. Malignant cells can be extracted from the tumor, irradiated in order to stop the proliferation, and engineered in order to produce growth factors. The modification involves a retroviral-mediated gene transfer. The growth factors produced by the modified cells can alert the immune system regarding the cancer and further attacking it [20].

## 4. Mechanism of Action of Checkpoint Inhibitors

The immune system is regulated by a complex balance between activation and inhibition of lymphocytes. Immune system has multiple cells such as T cells, B cells, and natural killer cells that function as cancer fighters with the T cells being the main effector and regulator cell. T cells present some specific receptors that function as activators while others as inhibitors of the T cell's activity.

Checkpoint inhibitors are molecules that permit activation of T lymphocytes through inhibiting the connection between a receptor that works as an inhibitor and its ligand. In this way, the checkpoint inhibitors allow the immune system to improve the efficacy of the fight against malignant cells. Thus, it can be concluded that immune checkpoints play a central role in maintaining an immune tolerance by inhibiting the immune system and so preventing the appearance of autoimmune phenomenon. In case of their blockage, a boost of the immune system will be observed, which can lead to a tumor control, explaining thus the efficacy of antibodies against checkpoint inhibitors in treating cancer [3].

TIM-3 is a protein that is part of the TIM family and has a role in regulating the function of Th1 lymphocytes. Overexpression of TIM-3 was observed to be associated with a poor prognosis in some forms of cancer [21].

**Figure 1:**
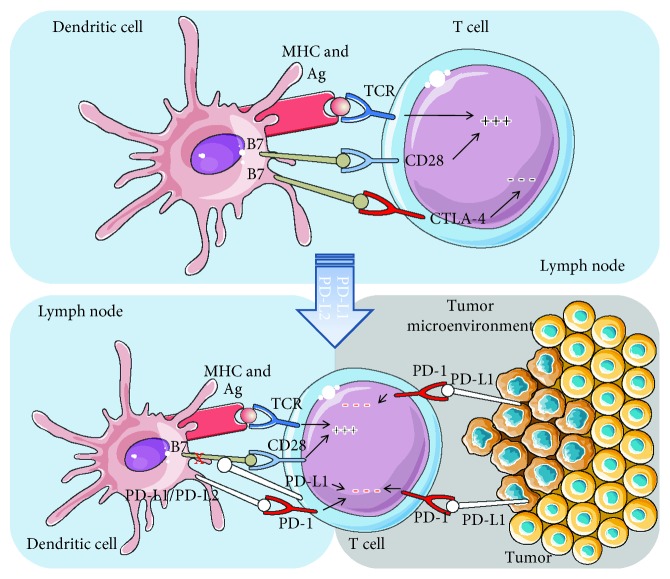
CTLA-4 and PD-1/PD-L1 pathways.

Two of the most important checkpoint inhibitors are considered to be CTLA-4 and PD-1, which will be described in the following paragraphs and illustrated in Figure 1.

### 4.1. CTLA-4 Pathway

The normal process following the activation of T cell leads to an upregulation of CTLA-4 (cytotoxic T lymphocyte antigen-4) on the surface of the T cell. CTLA-4 function is to be a downregulator of the T cell by outcompeting CD28 for B7 ligand, preventing in this way costimulation of the T cell and also by inducing T cell cycle arrest [22–25].

CTLA-4's affinity for B7 is higher than that of CD28. When CD28 binds to B7, it leads to a stimulatory signal, while CTLA-4 link to B7 will determine inhibitory signals [26]. Thus, the balance between the CLTA-4/B7 link and CD28/B7 link has to be carefully maintained. An imbalance in those connections can determine whether the T cell will activate or will undergo into an anergy state [27].

CTLA-4 is an important negative regulator in maintaining a normal immunologic environment. This was demonstrated by the fact that mice deficient in CTLA-4 suffered a CD28-dependent expansion of T cells in lymphatic organs. This proliferation led to death in less than 4 weeks postbirth due to lymphoproliferation [28].

Rudolph et al. noticed that blocking CTLA-4 will lead to a qualitative modification of the T memory cells. At the same time, a decrease in the number of CD4+ T cells and in this way a reduction in producing IFN-*γ*, IL-2, and tumor necrosis factor-alpha (TNF-*α*) as a response to the antigenic exposure was observed [29].

Pedicord et al. noticed that administration of anti-CTLA-4 antibodies will lead to a rise in the number of the CD8+ T cells with memory, leading to an increase in the secretion of TNF-*α* and IFN-*γ* [30].

On the basis of the preclinical studies, ipilimumab and tremelimumab were developed as anti-CTLA-4 antibodies. They function by blocking the interaction between CTLA-4 and B7, thus facilitating the linking between CD28 and B7, and leading to proliferation and activation increase of the T lymphocytes. Their action will lead to an increase in the antitumor immune response [31, 32].

Ribas et al. showed in a phase III study that the efficiency of tremelimumab was lower than that of chemotherapy with dacarbazine/temozolomide in patients with melanoma in advanced stages. Thus, not showing a statistically significant increase in overall survival rate, it was considered to be unfit for further studies [33].

On the other side, ipilimumab was discovered to significantly increase the overall survival rate in patients with advanced melanoma. Ipilimumab is a fully human IgG1 monoclonal antibody that inhibits the CTLA-4 binding to B7, and it was approved by FDA and EMA and is currently introduced in stage IV melanoma therapy.

Several combinations are ongoing in clinical trials to increase ipilimumab efficacy. Hodi et al. developed a clinical trial involving 676 patients with advanced melanoma whose disease progressed after treatment with at least one standard therapy. The patients were divided into 3 groups: the first group received ipilimumab in association with a vaccine (a gp100 peptide), the second group received ipilimumab only, and the third group received only the gp100 vaccine. When comparing the overall survival rate of the first group with that of the third group, a statistically significant increase was observed for the first group (10 months vs. 6.4 months). The same significant difference was noticed when confronted the data for the second group and the third group, 10.1 months vs. 6.4 months. Also, it was observed that the ORR (overall response rate) was the highest for the second group (10.9%), being almost double compared to the first one (5.7%), while the third group had an ORR of 1.5%. The longest duration of response was recorded in the second group, 11.5 months. Thus, the conclusion of the study was that ipilimumab can greatly increase the overall survival rate and response rate [34].

Wolchok et al. developed a clinical study involving 2 types of patients with metastatic melanoma: treatment-naïve and previously treated with chemotherapy. When looking at the survival rate at 4 years, it was discovered that patients therapeutically naïve had a more durable and significant survival rate in comparison to those who previously received chemotherapy [35].

A study developed by Prieto et al. has suggested that an association between ipilimumab and a high-dose IL-2 can have greater results than ipilimumab alone [36].

Looking to the survival rates in the study performed by Schadendorf et al. at both therapeutically naïve and experimented patients, a promising increase was observed compared to chemotherapy. This offers high hopes for patients with advanced melanoma [37].

Thus, ipilimumab has a long-term curative and regressive potential for advanced melanoma. Even though this molecule has serious adverse effects, if it is well monitored and treated promptly and correctly, it can be kept under control.

### 4.2. PD-1/PD-L1 Pathway

PD-1 (programmed death-1) receptor is a downregulator of the T cells, reducing the activity of T lymphocytes when binding to PD-L1 or PD-L2 ligands. PD-L1 is a receptor found on the plasmatic membranes of the malignant cells, while PD- L2 can be found on the surface of dendritic cells. The link between PD-1 and its ligand will inhibit kinase signaling pathways, thus leading to inactivation of the T cell. Using this mechanism of inhibiting T lymphocytes, tumor cells are able to escape the immune system and thus survive and develop further [38, 39]. Thus, it can be said that PD-1 blockade acts in the effector phase of the T cells. PD-L2 is a protein that can bind to PD-1, which has the ability to deplete T cells that present PD-1 on their surface [40].

PD-1 molecule serves as a negative regulator of the immune response, maintaining the self-tolerance of the organism. Early studies have shown that mice deficient in PD-1 developed autoimmune diseases such as lupus-like arthritis and glomerulonephritis and autoimmune dilated cardiomyopathy [41, 42].

PD-1/PD-L1 blockade leads to a successful antimalignant immune response. This involves the activation and proliferation of antigen-experienced T cells located at the malignant site [43, 44].

In order to generate CD8+ T cells that are tumor-reactive, an efficient presentation of the tumor antigens by APC is needed. A receptor found on the membrane of the T cell will recognize this tumor antigen, leading to initiation of activation of the T cell. The full activation of the T cell will happen only after the linkage between CD28 receptor and B7 ligand found on the surface of antigen-presenting cells [45].

Tumor-specific CD8+ T cells subsequently differentiate into effector T cells, undergo clonal expansion, traffic to the tumor microenvironment, and ultimately kill tumor cells displaying tumor-associated antigen, via release of several cytolytic effector molecules among which are granzyme A/B and perforin. For long-term immunologic memory and presumably durable disease control, a subset of effector T cells must differentiate into effector memory T cells, under the guidance of CD4+ helper T cells and dendritic cells. These are maintained for life and respond to rechallenge with antigen.

Based on the preclinical studies, a number of antibodies targeting the inhibition of linkage between PD-1 and PD-L1 entered clinical development. In this review, we will focus only on two antibodies that target PD-1 molecule: nivolumab and pembrolizumab, which have shown significant increase in the response rate in patients with advanced malign tumors [46, 47].

Nivolumab is a fully human IgG4 antibody directed against PD-1 receptor. By blocking the binding of PD-1 to PD-L1, this antibody will restore the natural tumor-specific immune response. It is recommended for patients with advanced melanoma and disease progression under ipilimumab and BRAF inhibitors, in the case of the patients with BRAF mutation. BRAF is a humane protoncogene that encodes a protein named B-Raf which is involved in a signalizing cascade with roles in growth promotion and cellular proliferation and differentiation.

Prior to approval, Weber et al. elaborated a phase III clinical study to compare the efficacy of nivolumab versus chemotherapy. In this way, the patients were divided into 2 groups: one that received nivolumab in monotherapy and the other one dacarbazine with carboplatin. The conclusion of the study was that nivolumab had a greater response rate and fewer adverse effects and made the treatment of adverse effects easier than chemotherapy [48].

In numerous phase III studies, nivolumab has proven a survival advantage when compared to the conventional treatments including chemotherapy. This was valid for both therapy-naïve or experimented patients [47–50].

Nivolumab can be administered in doses of 3 mg/kg every 2 weeks, and recently a new dosing schedule of 480 mg every 4 weeks it has been established. Indications for the new schedule of nivolumab can be found in Table 1. Recent studies have shown that nivolumab 480 mg every 4 weeks has the same results in terms of overall survival rate and response rate as nivolumab 3 mg/kg every 3 weeks, and also the safety profile is considered to be similar [51].

Pembrolizumab is an engineered humanized IgG4 monoclonal antibody which acts against the PD-1 (programmed death) receptor. Thus, pembrolizumab binds to PD-1 receptor and blocks its interaction with PD-L1 or PD-L2. As a result, the antitumor immunity will be reactivated by enhancing T cells to produce several activating cytokines such as IL-2, IL-6, IL-17, IFN-gamma, and TNF-alpha.

It can be administered in doses of 2 mg/kg every 3 weeks or 10 mg/kg every 2 or 3 weeks. The recommended dose sustained by the clinical studies found in the literature is demonstrated to be 2 mg/kg every 3 weeks, an increased not being associated with additional clinical benefit [52].

Pembrolizumab is recommended to be administered until the progression of the disease is confirmed or unacceptable toxicity is observed. Atypical responses at the treatment have been noted such as an initial increase in tumor size or the appearance of new lesions of small dimensions in the first months after the initiation of treatment, followed by a decrease in tumor size.

KEYNOTE 001 is a phase 1 trial with the purpose of assessing the appropriate dose of pembrolizumab in patients with progressive, locally advanced, or metastatic melanoma unable to respond at local therapy and with ECOG (Eastern Cooperative Oncology Group performance status) < 2 who could be ipilimumab naïve, treated, or refractory. The conclusions were that the longest median progression-free survival was obtained in the group treated with 2 mg/kg every 3 weeks when discussing about naïve patients, while for treated or refractory, there was no significant difference between the dosages [53–55].

75–83% of the patients treated with pembrolizumab experienced treatment-related side effects, but the majority of them had grade 1 or 2 in severity. Most common side effects related to the pembrolizumab treatment were fatigue, diarrhea, nausea, arthralgia, rash, and pruritus, out of which the first three were more common in patients receiving 10 mg/kg every 2 weeks in comparison with those who received 2 mg/kg every 3 weeks [54].

Pembrolizumab is frequently associated with immune adverse reactions. Most of them, including severe side effects, were remitted after the initiation of adequate medical treatment or stopping of pembrolizumab. The most frequent immunologic side effects were hypothyroidism, pneumonitis, and hyperthyroidism, followed by less common side effect such as colitis, hypophysitis, hepatitis, nephritis, and infusion-related reactions. Immunosuppressants can be used during the treatment in case of appearance of an immunologic side effect, but patients should avoid using them before starting the treatment with pembrolizumab because corticosteroids can alter the pharmacodynamics of pembrolizumab [54].

Ribas et al. showed in a clinical trial a response rate at 3-year of 33% for patients treated with pembrolizumab. 70–80% out of patients who initially responded maintained a clinical response over the 3-year period [56].

Literature studies showed that association immunotherapy can increase even further the response rate in comparison to one type of monoclonal antibody alone. The downside of this association is the increase in the number of the side effects and their severity. Thus, the increase in response rate and the severity of the side effects further have to be put in balance in order to pursue this therapy in further clinical trials [57, 58].

In Table 2, we resume the main side effects of checkpoint inhibitor therapy with the corresponding management for every grade of toxicity.

## 5. Resistance at Checkpoint Inhibitors

Recent clinical studies have shown that immune therapy can lead to resistance development. Thus, the population receiving this therapy can be divided into 3 groups: responders, innate resistance, and acquired resistance. Responders are patients that answer initially and maintain this answer. Innate resistance characterizes the patients that fail to respond from the first dose; thus, in this case the therapy should be halted and changed. Acquired resistance is shown in patients that first answer to the therapy, but after some cycles, they start to stop responding and eventually display disease progression [43, 44, 59]. Thus, it is a great and important therapeutic challenge to define and differentiate the responders and nonresponders, especially given the heterogeneity in patterns of response that can be seen with immune checkpoint inhibitors.

Failure of response to immune checkpoint inhibitors can arise from the following alterations: insufficient generation of antitumor T cells, inadequate function of tumor-specific T cells, and impaired formation of memory T cells. All of these alterations will lead to an imbalance between protumor state and antitumor state, with an increase in the protumor state.

Also, lack of sufficient or suitable tumor antigens, inadequate tumor antigen processing, or impaired presentation of tumor antigens can all lead to impaired formation of tumor-reactive T cells.

## 6. Side Effects of Checkpoint Inhibitors

We have put together the main side effects developed upon checkpoint inhibitors and the management of these side effects (Table 2).

## 7. Conclusions

In spite of an ascending trend in the incidence of melanoma, most cases are diagnosed in initial stages during which surgery is curative for a great majority of them. In the case of patients with high risk of developing metastasis, immunotherapy can be used after surgical excision. Thus, the greatest challenge seems to remain the disseminated type of melanoma, which seems to be the target for checkpoint inhibitors.

Immunotherapy with checkpoint inhibitors has a great potential in changing the face of oncologic treatments and so the outcomes of cancers are hard to treat until now. Numerous clinical studies proved great efficiency of checkpoint inhibitors regarding progression-free rate and overall survival rate in treating a variety of solid tumors (such as melanoma, non-small-cell lung carcinoma, and renal cell carcinoma) and hematologic cancers. Unfortunately, not all the studies confirm these results. Thus, it can be concluded that immunotherapy has limitations in cancer treatment. A further step that should be followed is the identification of biomarkers which can indicate when is best to use the checkpoint inhibitors.

## Figures and Tables

**Table 1:** Indications for administration of nivolumab 480 mg every 4 weeks [51].

Indications—nivolumab 480 mg every 4 weeks
(i) Metastatic melanoma (ii) Previously treated NSCLC (iii) Advanced renal cell carcinoma treated with prior antiangiogenic therapy (iv) Locally advanced or metastatic urothelial carcinoma which was previously treated, with progression during or after platinum-based chemotherapy (v) Classical Hodgkin lymphoma following relapse/progression after autologous hematopoietic stem cell transplantation (HSCT) and brentuximab vedotin or three or more lines of systemic therapy that includes autologous HSCT (vi) Recurrent/metastatic squamous cell carcinoma of the head and neck following platinum-based therapy (vii) Hepatocellular carcinoma after prior sorafenib therapy (viii) Adjuvant therapy for patients with completely resected melanoma with lymph node involvement or metastatic disease

**Table 2:** Adverse effects of checkpoint inhibitors [60].

Type of toxicity		Management of adverse effects
Cutaneous	Rash/inflammatory dermatitis	Rashes that can be controlled through topical treatments and oral antihistamines do not require stopping the immune therapy, but in the case of severe or unmanageable rashes, it is necessary to hold the therapy until the resolution of skin toxicity.
	Bullous dermatoses	If the blisters cover more or less than 10% of body surface area and do not affect the quality of life, the recommended treatment is topical corticosteroids. If the surface involved is more than 10%, the mucosal membranes are involved, and the lesions affect the quality of life, the immune therapy must be halted and continued only after skin resolution.
	Severe cutaneous adverse reactions (Stevens-Johnson epidermal necrolysis, acute generalized exanthematous pustulosis)	In case of maculopapular exanthem covering 10–30% of BSA (body surface area) in association with systemic symptoms, lymphadenopathy, or facial swelling, it is recommended to hold the checkpoint inhibitor therapy and give topical emollients, oral antihistamines, and topical corticosteroids with medium to high potency.
	Drug-induced hypersensitivity syndrome/drug reaction with eosinophilia and systemic symptoms (DHIS/DRESS)	

Pulmonary	Pneumonitis (identified on CT imaging as focal or diffuse inflammation of the lung parenchyma)	If the inflammation involves more than one lobe, but is less than 50% of the total parenchyma, the therapy is withheld until the resolution of symptoms, and prednisone 1–2 mg/kg/day is administered. If the inflammation involves more than 50% of the lung parenchyma or severe symptoms are present, the treatment will be permanently discontinued and antibiotics and systemic corticosteroids will be administered.

Renal	Nephritis	In case of G1 toxicity (creatinine 1.5–2 times over the baseline), only monitorisation is required. G2 toxicity (creatinine 2–3 times above baseline) leads to the hold of therapy, and if no improvement is observed, systemic corticosteroids will be administered (prednisone 1–2 mg/kg/day or equivalents). Grade 3 toxicity (creatinine > 3x baseline) leads to permanent discontinuation of therapy. Grade 4 toxicity has indication for dialysis and also administration of corticosteroids.

Hematologic	Autoimmune hemolytic anemia	Grade 1 toxicity allows continuation of therapy and a close clinical check-up. Grade 2 needs holding therapy and also administration of 0.5–1 mg/kg/d prednisone. Grade 3 or 4 requires permanent discontinuation, with administration of prednisone 1–2 mg/kg/d and supplementation with folic acid 1 mg daily. In case of grade 4 toxicity, if no improvement is observed, initiation of immunosuppressive drugs is required (rituximab, IVIG, cyclosporin A, mycophenolate mofetil).
	Acquired thrombotic thrombocytopenic purpura	All grades need therapy holding and hematology consult. G1 and G2 require the administration of 0.5–1 mg/kg/d prednisone, while grade 3 or 4 needs administration of methylprednisolone 1 g iv daily for 3 days, taking into consideration rituximab.
	Hemolytic uremic syndrome	Grades 1 and 2 does not require stopping the therapy, while grades 3 and 4 require the stop and initiation of exulizumab therapy 900 mg weekly for 4 doses, 1200 mg week5, then 1200 mg every 2 weeks.
	Aplastic anemia	Grade 1 requires therapy hold and administration of growth factor with close clinical observation. In case of grade 2 toxicity, it is added ATG (antithymocyte globulin) and cyclosporine administration to the protocol for grade 1. Patients with grade 3 or 4 have the same management as those with grade 2. If no response is observed, it is needed to repeat immunosuppression with ATG, cyclosporine, and cyclophosphamide. In the case of refractory patients, eltrombag needs to be taken into consideration.
	Lymphopenia	The only situation that requires holding therapy is a grade 4 toxicity (<250 PB lymphocyte count). In this case, it has to be initiated mycobacterium avium complex prophylaxis and Pneumocystis jirovecii prophylaxis and also cytomegalovirus (CMV)/human immunodeficiency virus (HIV)/hepatitis screening.
	Immune thrombocytopenia	Patients with a platelet count < 100/mcL (grade 1) need to continue the therapy with close clinical and laboratory evaluation. A count less than 75/mcL requires therapy holding with administration of oral prednisone 1 mg/kg/day 2–4 weeks, with taper over 4–6 weeks and also IVIG in case a faster increase in the platelet count is needed. Grade 4, meaning a platelet count < 25/mcL, is treated with prednisone 1–2 mg/kg/day and association with IGIV. In case of no response, rituximab or thrombopoietin receptor agonist can be used.
	Acquired hemophilia	G1 toxicity (5–40% of normal factor activity in the blood) needs holding of therapy and administration of 0.5–1 mg/kg/day prednisone. G2 toxicity (1–5% of normal factor activity in the blood) requires holding the therapy, administration of factor replacement, and administration of 1 mg/kg/d prednisone and 375 mg/m2 rituximab weekly for 4 weeks and/or cyclophosphamide 1–2 mg/kg/day. In case of severe symptoms (G3 or 4, <1% of normal factor activity in the blood), permanent discontinuation of therapy is required, in association with administration of bypassing agents (factor VII, factor VIII inhibitor bypass activity) and also administration of 1 mg/kg/d prednisone and 375 mg/m2 rituximab weekly for 4 weeks and/or cyclophosphamide 1–2 mg/kg/day. In case of bleeding, transfusions are needed.
